# Synthesis of Submicron-Sized TiB_2_ Powders by Reaction of TiC, B_4_C, and Ca in Molten CaCl_2_

**DOI:** 10.3390/ma18040744

**Published:** 2025-02-08

**Authors:** Ya-Long Wang, Guo-Hua Zhang

**Affiliations:** State Key Laboratory of Advanced Metallurgy, University of Science and Technology Beijing, Beijing 100083, China; wyl15613263509@163.com

**Keywords:** titanium diboride, molten salt, carbothermic reduction, boronization reaction

## Abstract

Submicron-sized TiB_2_ powders (300 nm–1 μm) were prepared by the reaction of TiC, B_4_C, and Ca assisted by molten CaCl_2_. The optimal reaction procedure (1200 °C and 25 wt.% CaCl_2_ + 25 wt.% Ca) was obtained by exploring the effects of the boronization reaction temperature and the addition of an amount of CaCl_2_. It was found that the introduction of CaCl_2_ not only promoted the reaction but also effectively inhibited the volatilization of excess Ca. Furthermore, SEM images of the products showed that the morphology and particle size of TiB_2_ were inherited from the carbothermal reduction product TiC, which was dominated by the “template/growth” mechanism. The process of the boronization reaction was that B atoms migrated from B_4_C and replaced the C atoms in the lattice of TiC.

## 1. Introduction

Recently, diborides have attracted significant attention in research in a multitude of areas. Among them, titanium boride (TiB_2_) has great potential in the fields of impact-resistant armor, cutting tools, conductive coatings, and cathode materials because of its excellent properties, including a low density (4.52 g/cm^3^), an outstanding elastic modulus (525 GPa), a high melting point (2980 °C), a high hardness (25–34 GPa), and remarkable electrical conductivities (6.25 × 10^5^ S/cm) [1,2,3]. The key to achieving the extensive applications of TiB_2_ material is the synthesis of powders. Nowadays, several preparation methods have been proposed to fabricate TiB_2_ powders, including carbo/borothermal reduction [4], self-propagating high-temperature synthesis (SHS) [5], the sol–gel method [6], and the molten salt-assisted method [7]. However, each method has its own unique limitations or shortcomings in the preparation process. For example, although the carbo/borothermal reduction method exhibits the advantages of low raw material costs and easy realization of large-scale production, it requires a relatively high temperature as well as a long reaction time, which inevitably leads to the grain growth of the TiB_2_ powders [8]. In addition, due to the strongly exothermic process of SHS, the safety of the reaction system cannot be ignored [9]. Although the sol–gel method can effectively prepare ultra-fine and uniformly distributed powder materials, the complexity of the process and the effective removal of residual carbon from the product are still problems restricting the wide application of this method [10]. Therefore, exploring a more economical, efficient, and safe preparation method to synthesize fine-grained TiB_2_ powders has important scientific and practical value.

Combining the economic advantage of the carbo/borothermal reduction method, a method to prepare TiB_2_ powders based on the reaction of TiC, B_4_C, and Ca was proposed in our previous work [11]. TiO_2_ was first reacted with C to synthesize TiC through carbothermal reduction (Equation (1)), and then the prepared TiC was reacted with B_4_C and excess Ca to produce the target product TiB_2_ (Equation (2)). Finally, the by-product CaC_2_ and the residual Ca could be efficiently removed by a subsequent leaching process. This method cleverly solves the volatility problem of B_2_O_3_ in the high-temperature process, which ensures the precise addition of boron. Additionally, the calculated heat released during the entire reaction process shows that this method is mild and controllable [12]. However, although the addition of excess Ca can promote the reaction, excess Ca volatilizes at high temperatures and deposits in the sealing area of the crucible as well as the wall of the furnace tube, which seriously impedes the continuous production process. Moreover, the excess calcium releases more heat during the leaching process and leads to the oxidation of the TiB_2_ powder. Thus, reducing the added amount of Ca is very necessary.

(1) $${\mathrm{TiO}}_{2}+3C=\mathrm{TiC}+2\mathrm{CO}$$

(2) $${4\mathrm{TiC}+2B}_{4}{C+3\mathrm{Ca}=4\mathrm{TiB}}_{2}{+3\mathrm{CaC}}_{2}$$

Molten salt can accelerate the mass transfer process between the reactants, reduce the synthesis temperature and particle size of the product, and even influence the morphology of the product [13,14,15]. In this work, a molten salt-assisted approach was adopted, with the specific strategy of replacing the half mass of the calcium (Ca) in the original reaction system with calcium chloride (CaCl_2_). The effect of the introduction of CaCl_2_ on the target chemical reaction and the product properties was discussed. Moreover, the mechanisms of the boronization reaction were also analyzed.

## 2. Materials and Methods

Commercially available TiO_2_ (purity > 99%, with a particle size of 100 nm, HushiLaboratoryReagent Co., Ltd., Shanghai, China), carbon black (purity > 98.5%, with a particle size of 24 nm, Mitsubishi Chemical Co., Ltd., Tokyo, Japan), B_4_C (purity > 99%, with a particle size of 2–4 μm, Aladdin Reagent Co., Ltd., Shanghai, China), and calcium (purity > 99.5%, with a particle size of 1–5 mm, BeijingXingRongYuanTech Co.,Ltd, Beijing, China) were used as starting materials, and CaCl_2_ (HushiLaboratoryReagent Co., Ltd., Shanghai, China) was added to form a molten-salt medium.

**Figure 1:**
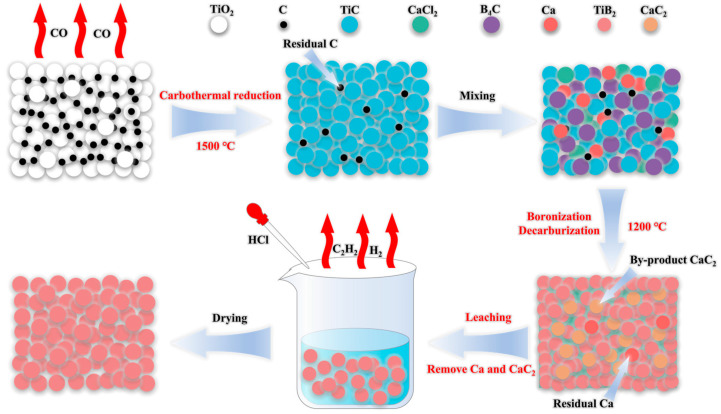
Schematic illustration for preparation of TiB_2_ powders.

First, TiO_2_ and C were mixed in the agate mortar for 30 min with the stoichiometric ratio (Equation (1)). Then, the mixture was charged into the graphite crucible and placed into a vertical furnace. The temperature of the carbon reduction reaction was set to 1500 °C and held for 4 h. After the reaction was completed, it was naturally cooled to room temperature in the furnace to obtain the target product titanium carbide (TiC). Subsequently, TiC and B_4_C were ball-milled for 4 h in a steel jar with steel balls, and ethanol was used as the medium. After milling, the mixed slurry was dried and mixed with Ca and CaCl_2_, and the mass ratio of TiC + B_4_C, Ca, and CaCl_2_ is provided in Table 1. To investigate the effect of temperature on the reaction, the specific mass ratio of Ca to CaCl_2_ was selected to be 1:1, and the total mass of Ca and CaCl_2_ was equal to the total mass of TiC and B_4_C (1000-0.5Ca-0.5CaCl_2_, 1100-0.5Ca-0.5CaCl_2_, and 1200-0.5Ca-0.5CaCl_2_). To investigate the effect of the added amount of CaCl_2_, the addition amount of Ca was half the total mass of TiC and B_4_C, while the content of CaCl_2_ was changed from 0, 0.5, and 1 times the total mass of TiC and B_4_C (1200-0.5Ca-0CaCl_2_, 1200-0.5Ca-0.5CaCl_2_, and 1200-0.5Ca-1CaCl_2_). The system was heated from room temperature to 1000–1200 °C at a rate of 10 °C min^−1^ and held for 4 h in an argon atmosphere. Afterward, the as-synthesized products were leached with deionized water and HCl to remove the CaCl_2_, as well as the residual Ca and CaC_2_. Finally, the products were repeatedly rinsed with deionized water followed by drying in a vacuum oven at 90 °C. The process for the preparation of TiB_2_ powders is illustrated in Figure 1.

The phase compositions of the powders were characterized by X-ray diffraction analysis (XRD, TTR III, Rigaku Corporation, Tokyo, Japan, Cu-Kα radiation, λ = 1.54178 Å). The products were scanned in the angle range (2θ) from 10 to 90°, and the scanning rate was 10 °C min^−1^. An oxygen and nitrogen hydrogen analyzer (EMGA-830, HORIBA, Kyoto, Japan) was utilized to measure the residual oxygen content. The morphologies of the powders were evaluated using scanning electron microscopy (FE-SEM, ZEISS SUPRA 55, Oberkochen, Germany) and a transmission electron microscope (TEM, JEM-2100, Tokyo, Japan) equipped with energy-dispersive X-ray spectroscopy (EDX). The lattice parameters and proportions of the products were acquired by using the Full Prof suite software package (v.5, GSAS). The particle size distributions of the products were elevated by a laser diffraction particle size analyzer. The structure relaxation calculations were performed with the Vienna ab initio simulation package (VASP). The generalized gradient approximation (GGA) in the form of Perdew–Burke–Ernzerhof (PBE) was adopted. The plane wave cut-off energy was taken as 450 eV, and the k-points were set to 5 × 5 × 3. Each atom’s force was minimized to below 0.01 eV/Å through the comprehensive optimization of all structures.

## 3. Results and Discussions

### 3.1. Carbothermal Reduction

**Figure 2:**
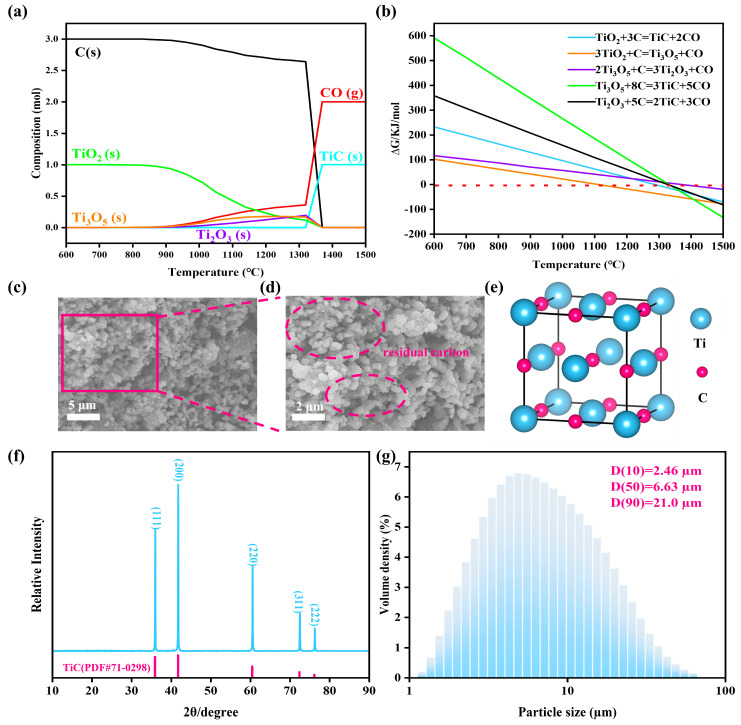
(**a**) Equilibrium product distribution of the carbothermal reduction between 600 °C and 1500 °C; (**b**) Gibbs free energy changes of reaction (1–5); (**c**) SEM images of the prepared TiC powder; (**d**) enlargement image of (**c**); (**e**) ball-stick representation of TiC; (**f**) XRD pattern of the prepared TiC powder; (**g**) laser particle size distribution of the prepared TiC powder.

As mentioned above, in the carbo/borothermal reduction reaction system, TiO_2_ reacted directly with B_4_C, which could produce an intermediate product (B_2_O_3_). It is worth noting that B_2_O_3_ is significantly volatile at high temperatures, leading to the loss of the boron source [16]. However, in this work, the pre-deoxygenation of the feedstock was achieved through a carbothermal reduction pretreatment step to reduce the evaporation of boron and consumption of Ca in the next step. Figure 2a and Figure 2b show the equilibrium product distribution of the carbothermal reduction and the Gibbs free energy changes of Equations (1) and (3)–(6), respectively. From a thermodynamic point of view, TiO_2_ could be fully converted to TiC when the temperature was higher than 1350 °C. Additionally, the carbothermal reduction did not directly proceed as expected according to Equation (1), while following the order TiO_2_ > Ti_3_O_5_ > TiC [17]. In order to ensure the reaction could be completed, the temperature of the carbothermal reduction was determined to be 1500 °C, and the SEM images of the synthesized TiC powder at 1500 °C are shown in Figure 2c and Figure 2d. The presence of the significant agglomeration of TiC particles can be clearly observed in Figure 2c, which is quantified in the laser particle size analysis results in Figure 2g. The particle size of the prepared TiC agglomerates was mainly distributed in the range of 6–10 µm, with a D (50) and D (90) of 6.63 and 21.0 µm, respectively. However, the particle size of the individual TiC particles was mainly distributed in the range of 300–800 nm. Additionally, although no diffraction peaks of impurities were found in the XRD pattern (Figure 2f), the presence of residual carbon in localized areas is marked in the circle in Figure 2d. Meanwhile, the oxygen content of the prepared TiC powder was 3.132 wt.%, which may have originated from the oxide layer of the TiC surface and trace residual oxides. The residual carbon could be removed by the reaction of Ca and C, and the residual oxides could also be converted into TiB_2_ during the boronization step [12].

(3) $${3\mathrm{TiO}}_{2}{+C=\mathrm{Ti}}_{3}O_{5}+\mathrm{CO}$$

(4) $${2\mathrm{Ti}}_{3}O_{5}{+C=3\mathrm{Ti}}_{2}O_{3}+\mathrm{CO}$$

(5) $${\mathrm{Ti}}_{3}O_{5}+8C=3\mathrm{TiC}+5\mathrm{CO}$$

(6) $${\mathrm{Ti}}_{2}O_{3}+5C=2\mathrm{TiC}+3\mathrm{CO}$$

### 3.2. Effects of Temperature and CaCl_2_ (Promotion of Thermodynamics as Well as Kinetics)

**Figure 3:**
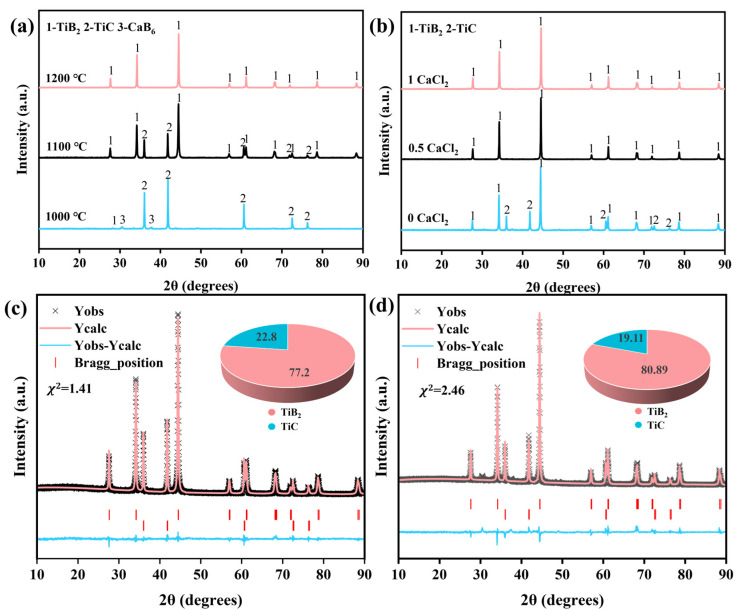
XRD patterns of products synthesized (**a**) at different temperatures and (**b**) with different addition amounts of CaCl_2_. (**c**) Rietveld-refined XRD results of 1100-0.5Ca-0.5CaCl_2_ products; (**d**) Rietveld-refined XRD results of 1200-0.5Ca-0CaCl_2_ products.

**Figure 4:**
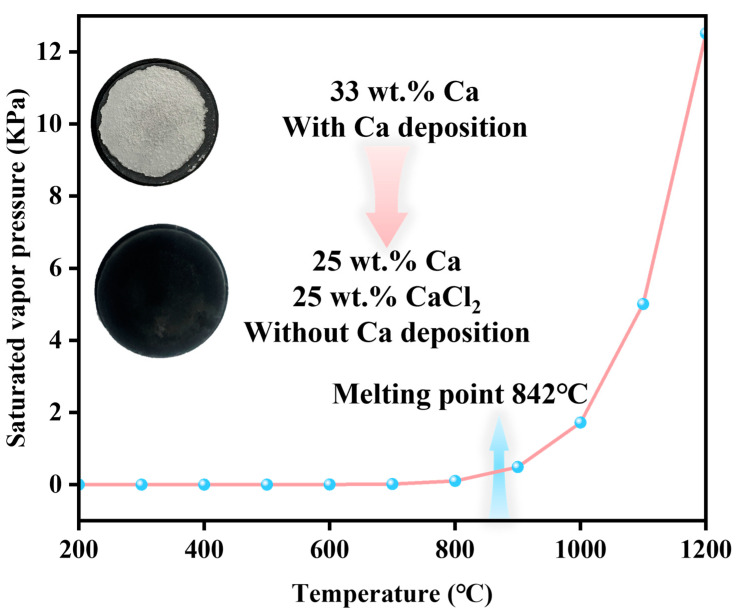
Saturated vapor pressure of Ca from 0 °C to 1200 °C, and internal surface of sealed crucible lid before and after introduction of CaCl_2_.

In this process, the carbothermal reduction was followed by a boronization reaction between the resulting product TiC and boron carbide. The effects of the temperature and the amount of added CaCl_2_ on the synthesized TiB_2_ powder will be discussed. The XRD patterns of the samples synthesized at different temperatures are shown in Figure 3a, where the addition amounts of CaCl_2_ and Ca were both 25 wt.%. At 1000 °C, the XRD showed that TiC was the major phase, while TiB_2_ and CaB_6_ were minor phases, indicating that the boronization reaction was difficult to occur at this temperature. The presence of CaB_6_ resulted from the reaction of Ca with B_4_C (Equation (7)). No characteristic peak of B_4_C was detected due to its weak diffraction peak intensity [18]. When the temperature was increased to 1100 °C, the main phase was changed to TiB_2_, and diffraction peaks of TiC were also observed. The Rietveld-refined XRD results of the 1100-0.5Ca-0.5CaCl_2_ products (Figure 3c) show that 77.2 wt.% TiB_2_ was formed, and the residual content of TiC was 22.8 wt.%. The absence of CaB_6_ may be due to the reaction between CaB_6_ and TiC, producing TiB_2_ and CaC_2_, which was verified in a previous work [12]. With the temperature increase to 1200 °C, single-phase TiB_2_ was obtained, indicating that the boronization reaction was completed. Thus, 1200 °C was necessary to ensure the reaction was completed. In addition, the XRD patterns of the products prepared at 1200 °C in the cases of different addition amounts of CaCl_2_ are shown in Figure 3b. When no CaCl_2_ was added, 80.89 wt.% TiB_2_ was formed, but 19.11 wt.% TiC was also retained, as shown in Figure 3d. Increasing the addition amount of CaCl_2_ to 25 wt.% and 40 wt.% produced no diffraction peaks of impurities except TiB_2_. Thus, the addition of CaCl_2_ is important to promote the boronization reaction. Although doubling the addition amount of Ca can achieve the same effect, it can also lead to significant calcium volatilization [12]. Figure 4 shows the saturated vapor pressure of Ca between 200 °C and 1200 °C based on Equations (8) and (9).

(7) $${7\mathrm{Ca}+6B}_{4}{C=4\mathrm{CaB}}_{6}{+3\mathrm{CaC}}_{2}$$

(8) Ca(l)=Ca(g)

(9) $$\Delta G=-RT\ln\frac{p}{p*}$$

where Δ*G* is the change in the Gibbs free energy of Equation (8); R is the molar gas constant (8.314); *T* is the temperature; *p* is the saturated vapor pressure of Ca; and p* is the standard atmospheric pressure. It can be observed that when the temperature was higher than the melting point of Ca (842 °C), the saturated vapor pressure of Ca rose sharply, and the excess Ca transformed into calcium vapor. Despite Ca being able to transfer via its vapor state, it was difficult to avoid its volatilization at high temperatures, and the volatilized calcium deposited on the lid of the sealed crucible, as shown in Figure 4. Deposited calcium on the crucible or furnace wall leads to an obstacle to the continuous production process and a safety risk. However, when 25 wt.% CaCl_2_ was added, the volatilization of Ca was greatly reduced, and the deposited Ca was almost invisible on the sealed crucible. In fact, metal can react readily with various negatively charged ions in the molten salt, leading to the dissolution of the metal [19]. Suzuki et al. reported that Ca could be supplied as vapor and saturate in the molten CaCl_2_ (about 6 mol% Ca at 1000 °C) [20]. The dissolution of Ca in CaCl_2_ reduces the activity of Ca and inhibits its volatilization. Additionally, the introduction of CaCl_2_ does not introduce new waste in the subsequent leaching process. In conclusion, CaCl_2_ played a crucial role in this work and was not only able to provide a molten liquid-phase environment for chemical reactions, thereby effectively facilitating the transfer and exchange of substances, but also had the ability to dissolve part of the calcium, which significantly reduced the volatility of Ca at high temperatures.

### 3.3. Morphology and Particle Size of Prepared TiB_2_ Powder

**Figure 5:**
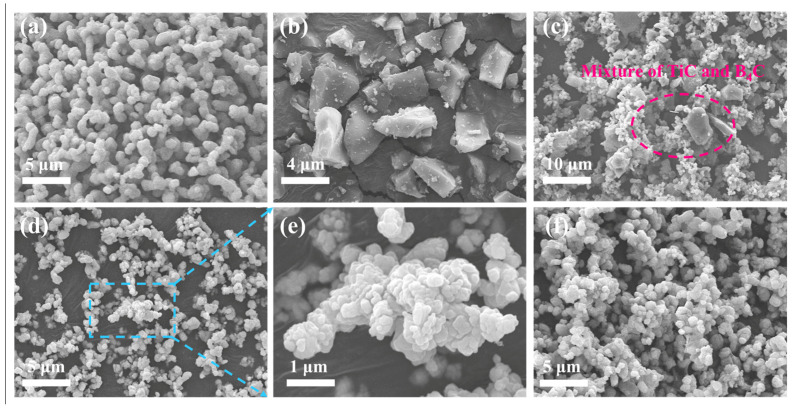
SEM images of reactants and products of boronization reactions: (**a**) TiC, (**b**) B_4_C, (**c**) mixture of TiC and B_4_C after ball milling, (**d**) TiB_2_-1200-0.5Ca-0.5CaCl_2_, (**e**) enlarged images of (**d**), and (**f**) TiB_2_-1200-0.5Ca-1CaCl_2_.

Figure 5 shows the SEM images of the reactants and products of the boronization reaction. Among them, Figure 5a and Figure 5b show the micro-morphologies of TiC and B_4_C, respectively. Figure 5c presents the distributions of TiC and B_4_C after ball-milling, where larger B_4_C particles were surrounded by smaller TiC particles. Then, 25 wt.% Ca and 25 wt.% CaCl_2_ were added manually due to the significantly different particle sizes. Subsequently, the morphological characteristics and particle size distribution of the resulting products after the boronization reaction at 1200 °C and the corresponding leaching step are shown in Figure 5d. TiB_2_ had an irregular shape, with particle sizes mostly between 500 nm and 1 μm, as further demonstrated by the enlarged view of the agglomerates in Figure 5e. However, after increasing the CaCl_2_ addition to 40 wt.%, the particle size and morphology did not change greatly, and it was found that the particle size prepared by this method was very close to that of the TiC. According to previous investigations, the molten-salt synthesis process always follows two mechanisms—the “template/growth” mechanism and the “dissolution/precipitation” mechanism—the selection of which is highly dependent on the solubility of the reactants in the molten-salt liquid-phase medium [21]. Liu et al. synthesized ultrafine TiB_2_ powders by using TiO_2_ powder, B powders, and NaCl/KCl. They found that TiB_2_ would precipitate from the molten salt to nucleate and grow when its concentration reached the supersaturation condition in the molten-salt medium, which was dominated by the “dissolution/precipitation” mechanism [7]. Considering that both TiC and B_4_C are covalent compounds with little solubility in CaCl_2_, the TiB_2_ products retain the morphology/size of the reactant TiC. The “template/growth” mechanism was expected to dominate the molten-salt route in this work [19], and the specific reaction mechanism will be discussed in the next section.

**Figure 6:**
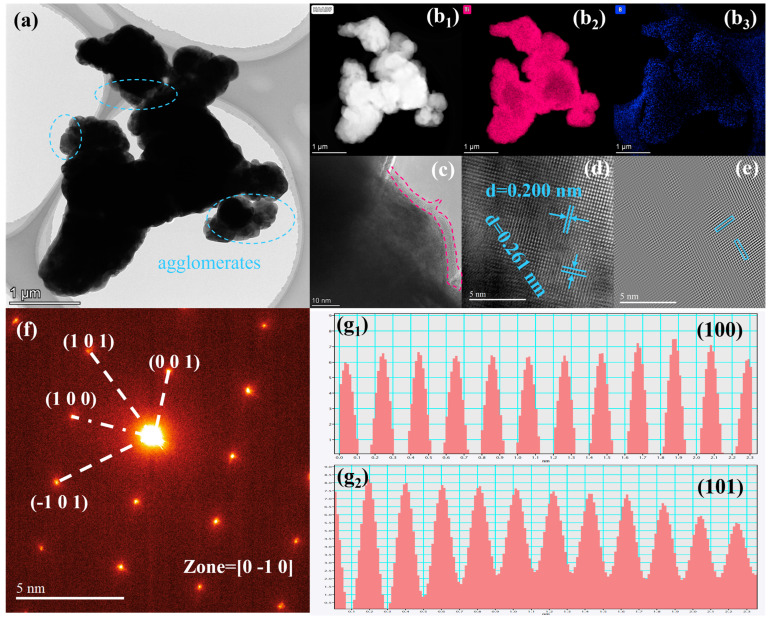
TEM images of prepared TiB_2_-1200-0.5Ca-0.5CaCl_2_ powders: (**a**) TEM image, (**b**) EDS mapping: (**b_1_**) HAADF image, (**b_2_**) Ti, (**b_3_**) B, (**c**,**d**) HRETEM image, (**e**) IFFT image, (**f**) SAED image, and (**g_1_**) interplanar distance image of (100) plane, (**g_2_**) interplanar distance image of (101) plane.

The representative microstructure of the TiB_2_ particles obtained with 25 wt.% Ca and 25 wt.% CaCl_2_ at 1200 °C was also characterized by TEM, as shown in Figure 6. The TEM images (Figure 6a) exhibit that the prepared TiB_2_ powder was severely agglomerated. Additionally, the size of the single particles ranged from 300 nm to 1 μm, which is consistent with the results of the SEM images. The atomic number of B is small, which produces a low accuracy of identification. Thus, the EDS of Ti and B can only semi-quantitatively indicate their distributions. An oxide layer with a thickness of about 3 nm was clearly displayed on the surfaces of the particles (Figure 6c). The presence of an oxide layer on TiB_2_ particles is a common phenomenon, which can reduce the specific surface energy of the powder and hinder the diffusion of atoms and, finally, affect the sintering densification process [7]. Additionally, the lattice spacing can be clearly observed in the HERTRM image of Figure 6d and was confirmed to be 0.2 nm and 0.261 nm, corresponding to the (101) planes and (100) planes of TiB_2_, respectively. The selected-area electron diffraction (SAED) pattern shows a series of clear and well-organized diffraction spots, which confirms that the TiB_2_ powder was well-crystalized.

### 3.4. Mechanism of Boronization Reaction

**Figure 7:**
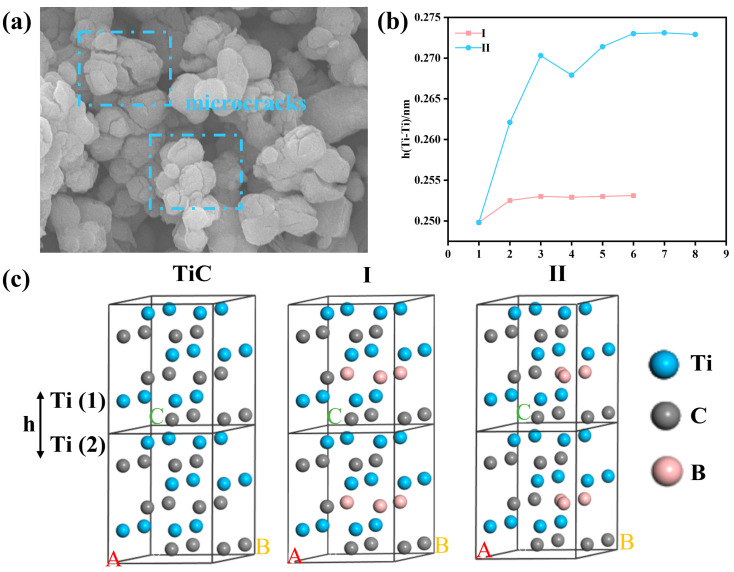
(**a**) Microcracks in prepared 1100-0.5Ca-0.5CaCl_2_ powders, (**b**) the change in the distance between neighboring Ti layers with N-step lattice relaxation simulation, and (**c**) TiC-based supercell with different B-doping methods.

In this work, the boronization reaction can be described as a process in which B atoms leave B_4_C through a mixture of molten-salt CaCl_2_ and Ca and diffuse to the surface of TiC to produce TiB_2_. TiC acts as a reactive template during the boronization process, and the morphology and particle size of TiB_2_ is inherited from TiC. Interestingly, as shown in Figure 7a, several microcracks appeared in the products of 1100-0.5Ca-0.5CaCl_2_, and the formation mechanism of these microcracks may have originated from the tensile stress effect of boron during its diffusion penetration into the TiC matrix. To explain this phenomenon, the microstress of TiB_2_-1100-0.5Ca-0.5CaCl_2_ and TiC were calculated using the Williamson–Hall method [22,23]:

(10) $$\beta cos\theta =\frac{0.9\lambda }{D}+\varepsilon \left(4\sin\theta \right)$$

where *D* is the grain size of the particles, and ε is the internal strain.

The microstress of the TiC and 1100-0.5Ca-0.5CaCl_2_ powders were calculated to be 2.04 × 10^−6^ and 2.34 × 10^−6^, respectively. It is obvious that the substitution of boron for C produced internal stress in the TiC powders. Furthermore, to verify the actual source of these microstresses, the first-principles calculations using the density functional theory (DFT) were performed. According to the report by Vekables et al., the only remaining “trace” concentration of boron in TiC was able to induce the precipitation of TiB_2_ in the direction of the (111) crystal plane of the TiC lattice [24]. Thus, a model of a supercell containing 24 atoms was designed, consisting of three closely spaced layers. Each layer was composed of Ti and C atoms arranged according to the (111) crystal plane orientation. Such a design was intended to simulate the penetration of boron into TiC and to observe its possible effects on the structure of the TiC. Generally, there are two different strategies for doping boron atoms into TiC supercells. The first strategy is that all three B atoms directly replace the original C atoms in the supercell, as shown in Figure 7c(I). The second strategy is that two of the B atoms take the place of C atoms, while the remaining third B atom is specially placed in an interstitial position corresponding to the TiB_2_ lattice, as shown in Figure 7c(II) [25,26]. The entry of boron atoms into the supercell causes lattice relaxation, and the simulation results of this process are displayed in Figure 7b. In the first case, after six iterations, three C atoms were replaced by three B atoms. It is observed that the equilibrium surface spacing between neighboring (111) Ti layers increased by 8% from 0.2498 nm to 0.2531 nm. In the second case, after eight iterations, the increase in the equilibrium surface spacing between the (111) Ti layers was more significant, reaching 10.5%, which may imply that the B atom at the interstitial position had a more drastic effect on the lattice structure of TiC. As a result, the increased spacing between the TiC (111) crystal planes promoted the diffusion of B and the formation of TiB_2_. The spacing of TiB_2_ was 28% larger than that of the TiC grain, resulting in large tensile stress and the cracking of the TiC grains, as well as the formation of new TiB_2_ grains. Furthermore, the atomic radius of B was 85 pm, which was larger than that of C (77 pm); meanwhile, the molar volume of TiB_2_ (15.41 cm^3^/mol) increased by about 26% relative to the molar volume of TiC (12.22 cm^3^/mol). A significant increase in the molar volume typically results in a volume expansion of the TiC lattice during the process of the boronization reaction.

**Figure 8:**
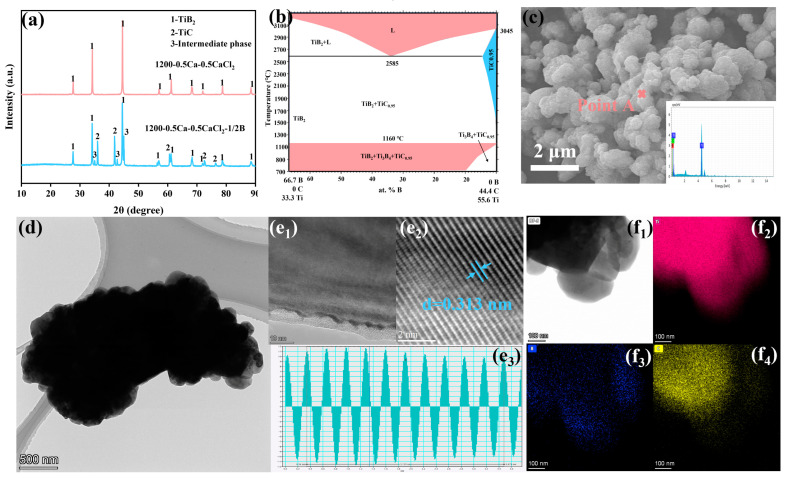
(**a**) XRD patterns of products of boronization reaction with different contents of added B_4_C; (**b**) phase diagram of TiC-TiB_2_, (**c**) SEM image and EDX spectrum of 1200-0.5Ca-0.5CaCl_2_-1/2B powder, (**d**) TEM characterization of 1200-0.5Ca-0.5CaCl_2_-1/2B particles, (**e1**,**e2**) HR-TEM image, (**e3**) interplanar distance, and (**f**) EDS mapping of 1200-0.5Ca-0.5CaCl_2_-1/2B particles: (**f_1_**) BF image, (**f_2_**) Ti, (**f_3_**) B, (**f_4_**) C.

Moreover, the effect of the varying amounts of the added B_4_C on the composition of the final product is also explored in this section. Figure 8a clearly demonstrates the differences in the XRD patterns of the products obtained after the addition of half-stoichiometric (1/2-stoichiometric) versus full-stoichiometric B_4_C, respectively. It is observed that the added amount of B_4_C did not reach the full-stoichiometric ratio. In addition to the expected TiB_2_ and TiC phases, the presence of diffraction peaks of an unknown phase was detected, which provides a new perspective for understanding the phase transformation mechanism in the boron penetration process. The binary TiC-TiB_2_ diagram obtained from Factsage 8.3 using the database of SpMCBN [27] shows that Ti_3_B_4_ was stabilized as an intermediate product in the transformation of TiC into TiB_2_ when the temperature was less than 1160 °C, and a further increase in temperature could promote the transformation of Ti_3_B_4_ into TiB_2_. However, Ti_3_B_4_ (57 at.% B) is a peritectic product between orthorhombic and metal liquid TiB_2_ at 2180 °C [28,29,30], which is difficult to generate under the presented conditions. Hence, in this work, we prefer to consider the observed phase as a prototypical TiB_2_ structure originating from the TiC transformation process rather than Ti_3_B_4_. Figure 8c shows an SEM image of the 1200-0.5Ca-0.5CaCl_2_-1/2B particles, and the morphology and particle size are consistent with the pure TiB_2_ particles prepared in this work. However, the EDX spectrum of point A demonstrates the simultaneous presence of the elements Ti, C, and B, with a mass ratio of Ti, B, and C of 56:30:14. This result shows that the C atoms in TiC were not completely replaced by B, and a prototypical structure close to TiB_2_ was formed. Although the lattice spacing of the edge particle (Figure 8(e_2_)) was determined to be 0.313 nm, which corresponded to the (001) crystal plane spacing of TiB_2_, the EDS mapping, as shown in Figure 8f, shows that the TiC particles contained traces of B and edge TiB_2_ particles contained residual C. These phenomena suggest the diffusion of B into TiC to substitute for C.

## 4. Conclusions

In summary, using TiC, B_4_C, Ca, and CaCl_2_ as raw materials, submicron-sized TiB_2_ powders were synthesized via the molten-salt method at 1200 °C. The conclusions are given below:

(1) The introduction of CaCl_2_ not only promoted the reaction but also reduced the volatilization of excess Ca.
(2) The particle size and morphology of TiB_2_ were inherited from TiC based on the “template/growth” mechanism, and the particle size of the prepared TiB_2_ ranged from 300 nm to 1 μm.
(3) Boronization was a process in which B atoms from B_4_C diffused into the TiC lattice and gradually replaced the C atoms.

## Figures and Tables

**Table 1:** The specific reaction parameters of the boronization reactions.

Sample No.	Temperature (°C)	Mass Ratio (wt.%)		
		TiC + B_4_C (Mixture of TiC and B_4_C)	Ca	CaCl_2_
1000-0.5Ca-0.5CaCl_2_	1000	50	25	25
1100-0.5Ca-0.5CaCl_2_	1100	50	25	25
1200-0.5Ca-0.5CaCl_2_	1200	50	25	25
1200-0.5Ca-0CaCl_2_	1200	66	33	0
1200-0.5Ca-1CaCl_2_	1200	40	20	40
1200-0.5Ca-0.5CaCl_2_-1/2B	1200	50 (molar ratio of TiC and B_4_C = 4:1)	25	25

## Data Availability

The original contributions presented in the study are included in the article, further inquiries can be directed to the corresponding author.
